# Thermal and Postural Effects on Fluid Mixing and Irrigation Patterns for Intraventricular Hemorrhage Treatment

**DOI:** 10.1007/s10439-022-03130-9

**Published:** 2023-01-21

**Authors:** Coskun Bilgi, Faisal Amlani, Heng Wei, Nick Rizzi, Niema M. Pahlevan

**Affiliations:** 1 Department of Aerospace and Mechanical Engineering, University of Southern California, Los Angeles, CA USA; 2 CentraleSupélec, ENS Paris-Saclay, CNRS, LMPS - Laboratoire de Mécanique Paris-Saclay, Université Paris-Saclay, 91190 Gif-Sur-Yvette, France; 3 Research and Development Department, IRRAS Inc., San Diego, CA USA; 4 Division of Cardiovascular Medicine, Keck School of Medicine, University of Southern California, CA Los Angeles, USA

**Keywords:** Computational fluid dynamics (CFD), Thermally-induced convection, Targeted delivery, Self-irrigating catheter, Ventricular drain, Flow in lateral ventricle

## Abstract

Intraventricular hemorrhage is characterized by blood leaking into the cerebral ventricles and mixing with cerebrospinal fluid. A standard treatment method involves inserting a passive drainage catheter, known as an external ventricular drain (EVD), into the ventricle. EVDs have common adverse complications, including the occlusion of the catheter, that may lead to permanent neural damage or even mortality. In order to prevent such complications, a novel dual-lumen catheter (IRRAflow®) utilizing an active fluid exchange mechanism has been recently developed. However, the fluid dynamics of the exchange system have not been investigated. In this study, convective flow in a three-dimensional cerebral lateral ventricle with an inserted catheter is evaluated using an in-house lattice-Boltzmann-based fluid–solid interaction solver. Different treatment conditions are simulated, including injection temperature and patient position. Thermal and gravitational effects on medication distribution are studied using a dye simulator based on a recently-introduced (pseudo)spectral convection–diffusion equation solver. The effects of injection temperature and patient position on catheter performance are presented and discussed in terms of hematoma irrigation, vortical structures, mixing, and medication volume distribution. Results suggest that cold-temperature injections can increase catheter efficacy in terms of dye distribution and irrigation potential, both of which can be further guided by patient positioning.

**Supplementary Information:**

The online version contains supplementary material available at 10.1007/s10439-022-03130-9.

## Introduction

The majority of hemorrhagic strokes happen due to intracerebral (ICH) or subarachnoid hemorrhages (SAH).^42^ These hemorrhages cause 13% of strokes in the United States, accounting for more than 100,000 patients per year.^42^ A common complication of ICH and SAH is intraventricular hemorrhage (IVH), where the hemorrhage leaks into the cerebral ventricles.^16,29,33^ Although IVH can vary in severity from occupying mild layers of blood to full ventricles, any presence of IVH resulting from other intracranial hemorrhages can be attributed to poor clinical outcomes.^17,29^ Patients with ICH/SAH + IVH components (two simultaneous hemorrhages) have more than a 50% 30-day mortality rate, and half of these deaths occur in the first 48 h.^10,17^ High mortality rates have been related to the blood volume in the cerebral ventricle and the presence of hydrocephalus.^38,49^ Even though these hemorrhages occur commonly, there is still controversy as to the appropriate method of treatment.^23^ Arguably, the most common and beneficial treatment involves draining the blood and cerebrospinal fluid mixture (*hematoma*) continuously with a passive external ventricular drain (EVD) in order to regulate the edema and hydrocephalus.^24,51^ While EVD placement may improve the outcome, it may lead to catheter-related complications.^21^ Even when the best EVD insertion and management guidelines are followed,^21^ patients still experience side effects due to the occlusion of the catheter.^19^ Frequently reported complications include secondary intracranial hemorrhage (28%), infection (23.2%), temporary catheter occlusion (42%), permanent catheter occlusion (18.6%), and replacement of the EVD (25%).^19^ Temporary occlusions are usually solved by displacing the blood clots *via* an injection of a small amount of saline through the catheter (a so-called “irrigation procedure”). However, this procedure lacks a well-defined clinical protocol and must be performed at the physician’s discretion.^19^

In order to take advantage of irrigation while maintaining sterility, a novel dual-lumen self-irrigating catheter system has been recently developed (IRRAflow®, IRRAS, Stockholm, Sweden). This dual-lumen catheter is connected to an intelligent digital cassette (feedback mechanism for peristaltic pumping) and powered by a control unit with a user interface. The catheter is designed to prevent the issues observed with conventional EVD usage by delivering periodic irrigation with drainage and continuous pressure monitoring (so-called *active fluid exchange*). Several clinical studies have illustrated the ability of this dual-lumen catheter system to deliver tissue Plasminogen Activator (tPA; a clot breaking agent) in a continuous manner while enabling quicker evacuation of blood in the ventricles while negating catheter occlusions^25,41^ (increasing EVD efficiency and decreasing complications^52^).

Although case studies with this catheter system (IRRAflow®) have documented the effectiveness of such self-irrigating catheters in shortening hospitalization times with no catheter-related complications,^25–28,36,37,41^ the underlying physics of the active fluid exchange mechanism and drug delivery is not yet fully understood. To the best of our knowledge, there is no study investigating the fluid dynamics of any type of EVD catheters nor their drug delivery mechanisms. Using a computational fluid dynamics approach (see Methodology section for the governing equations and numerical methods), this study investigates the fluid exchange system within the lateral cerebral ventricle at different fluid injection temperature and patient body position. The results of this study might provide insights into treatment procedures of active-irrigation catheters and, further, may contribute to improved patient care by elucidating optimized drug delivery patterns.

## Methodology

### Fluid–Solid Interaction (FSI) Solver

#### Lattice Boltzmann Method (LBM)

In order to simulate fluid flow, we used a lattice Boltzmann method (LBM) that combines discrete kinetic theory and modified molecular dynamics. Unlike conventional continuum-based methods, LBM is a mesoscopic method that behaves like a conservation equation solver for the corresponding macroscopic variables while providing a viable way for the inclusion of microscopic dynamics at the interfaces.^12^ This approach has been utilized for various fluid dynamics problems including those related to turbulent,^14^ multiphase,^40^ thermal,^31,39^ and bio-fluid^45,46^ flows.

In LBM, a particle distribution function ensures that fluid particles move synchronously on a regular lattice. The function is chosen to satisfy conservation equations, where the fluid is Galilean invariant and isotropic.^47^ In the present work, our model has two sets of distributions ${f}_{i}\left({\varvec{x}}, t\right)$ and ${g}_{i}\left({\varvec{x}}, t\right)$ for the flow and temperature fields, respectively, each representing the probability that a particle traveling with velocity ${{\varvec{e}}}_{{\varvec{i}}}$ occupies the position ${\varvec{x}}$ at a time $t$. Both ${f}_{i}$ and ${g}_{i}$ are governed by their respective Bhatnagar-Gross-Krook collision model^9^ with 19 discrete velocity vectors (D3Q19), given by

1 $$\begin{array}{c}{f}_{i}\left({\varvec{x}}+{{\varvec{e}}}_{{\varvec{i}}}\Delta t, t+\Delta t\right)-{f}_{i}\left({\varvec{x}}, t\right)=-\frac{1}{\tau }\left[{f}_{i}\left({\varvec{x}}, t\right)-{f}_{i}^{eq}\left({\varvec{x}}, t\right)\right]+\Delta t{F}_{i}+\Delta t{S}_{i},\, i=1,...,19,\end{array}$$

2 $$\begin{array}{c}{g}_{i}\left({\varvec{x}}+{{\varvec{e}}}_{{\varvec{i}}}\Delta t, t+\Delta t\right)-{g}_{i}\left({\varvec{x}}, t\right)=-\frac{1}{{\tau }_{T}}\left[{g}_{i}\left({\varvec{x}}, t\right)-{g}_{i}^{eq}\left({\varvec{x}}, t\right)\right],\, i=1,...,19.\end{array}$$

Here, $\Delta t$ and $\Delta x$ are the timestep and lattice spacing (spatial discretization), respectively; $c=\Delta x/\Delta t$ is the lattice speed (equaling unity in this study with a corresponding lattice sound speed of ${c}_{s}=c/\sqrt{3}$); ${S}_{i}$ is the buoyancy force flux; ${F}_{i}$ is a forcing term; and $\tau , {\tau }_{T}$ are dimensionless relaxation time constants respectively associated with fluid viscosity $\mu =\rho {c}_{s}^{2}(\tau -1/2)\Delta t$ (fluid density $\rho )$ and thermal diffusivity $\alpha ={c}_{s}^{2}({\tau }_{T}-1/2)\Delta t$. The local equilibrium distributions ${f}_{i}^{eq}$ and ${g}_{i}^{eq}$ are given by^39^

3 $$\begin{array}{c}{f}_{i}^{eq}= {\upomega }_{i}\frac{P}{ {{c}_{s}}^{2}}+{\upomega }_{i}\rho \left[\frac{{{\varvec{e}}}_{{\varvec{i}}}\cdot {\varvec{v}}}{ {{c}_{s}}^{2}}+\frac{{\left({{\varvec{e}}}_{{\varvec{i}}}\cdot {\varvec{v}}\right)}^{2}}{ 2{c}_{s}^{2}}-\frac{{{\varvec{v}}}^{2}}{ 2{c}_{s}^{2}}\right],\end{array}$$

4 $$\begin{array}{c}{g}_{i}^{eq}= {\upomega }_{i}T\left[1+\frac{{{\varvec{e}}}_{{\varvec{i}}}\cdot {\varvec{v}}}{ {{c}_{s}}^{2}}+\frac{{\left({{\varvec{e}}}_{{\varvec{i}}}\cdot {\varvec{v}}\right)}^{2}}{ 2{c}_{s}^{2}}-\frac{{{\varvec{v}}}^{2}}{ 2{c}_{s}^{2}}\right],\end{array}$$

for temperature$T\left({\varvec{x}}, t\right)$; velocity ${\varvec{v}}\left({\varvec{x}}, t\right)$**;** pressure $P\left({\varvec{x}}, t\right)$ is pressure; and weighting factors ${\omega }_{i}$ (equal to 1/3 for $i=1$; 1/18 for$i=2,...,7$; and 1/36 for the rest^46^). The buoyancy force density ${S}_{i}$ can be formulated by the Boussinesq approximation^31^ as

5 $$\begin{array}{c}{S}_{i}=\frac{{\omega }_{i}{\rho }_{0}\beta \left(T\left({\varvec{x}}, t\right)-{T}_{\infty }\right)}{{c}_{s}^{2}}{\varvec{g}}\cdot {{\varvec{e}}}_{{\varvec{i}}},\end{array}$$

where $\beta$ is the thermal expansion coefficient; ${\rho }_{0}=P/{c}_{s}^{2}$; ${T}_{\infty }$ is a reference temperature; and ${\varvec{g}}$ is the gravity vector. The terms ${F}_{i}$ are defined as

6 $$\begin{array}{c}{F}_{i}=\left(1-\frac{1}{2\tau }\right){\omega }_{i}\left(\frac{{{\varvec{e}}}_{{\varvec{i}}}-{\varvec{v}}}{{ {c}_{s}}^{2}}+\frac{\left({{\varvec{e}}}_{{\varvec{i}}}\cdot {\varvec{v}}\right)}{{{c}_{s}}^{4}}{{\varvec{e}}}_{{\varvec{i}}}\right)\cdot {\varvec{b}},\end{array}$$

where ${\varvec{b}}$ is the force in Eulerian coordinates. Macroscopic flow properties are calculated by

7 $$\begin{array}{c}P={{c}_{s}}^{2}\sum {f}_{i},\end{array}$$

8 $$\begin{array}{c}\rho v=\sum {{\varvec{e}}}_{{\varvec{i}}}{f}_{i}+\frac{1}{2}b\Delta t,\end{array}$$

9 $$\begin{array}{c}T=\sum {g}_{i}.\end{array}$$

#### Solid Domain and Finite Element Method

**FIGURE 1:**
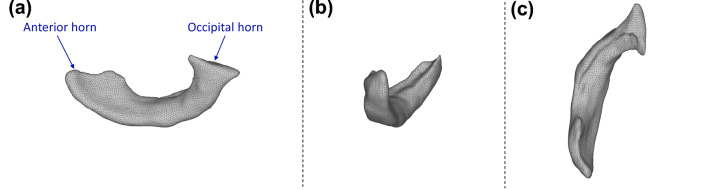
The smoothed cerebral lateral ventricle geometry and finite element mesh corresponding to (a) sagittal, (b) axial, and (c) coronal planes of the lateral brain ventricle considered in this study.

An anatomically realistic lateral brain ventricle is considered in this study.^6^ Three-dimensional (3D) rendered images of this geometry is acquired from a repository^43^ and subsequently separated using SOLIDWORKS® (Dassault Systèmes, France) to obtain the final form. MeshLab^13^ is used for smoothing the geometry and generating three-node triangular mesh elements (Fig. 1).

Although the ventricle geometry is a 3D object in the fluid domain, it can be reduced to a shell with the thin wall assumption. Hence, it is discretized as a 2D plate in Lagrangian coordinates, where the wall deformations can be modeled as

10 $$\begin{array}{*{20}c} {\rho _{s} h\frac{{\partial ^{2} {\varvec{X}}}}{{\partial t^{2} }} = \sum\limits_{{i,j = 1}}^{2} {\frac{\partial }{{\partial s}}\left[ {Eh\varphi _{{ij}} \left( {\delta _{{ij}} - \sqrt {\frac{{\partial {\varvec{X}}}}{{\partial s_{i} }} \cdot \frac{{\partial {\varvec{X}}}}{{\partial s_{j} }}} } \right)\frac{{\partial {\varvec{X}}}}{{\partial s_{j} }} - \frac{\partial }{{\partial s_{j} }}\left( {E_{I} \gamma _{{ij}} \frac{{\partial ^{2} {\varvec{X}}}}{{\partial s_{i} \partial s_{j} }}} \right)} \right] + {\varvec{B}}_{L} } ,} \\ \end{array}$$

where ${\rho }_{s}$ is the density of the solid wall; $h$ is the wall thickness; ${\varvec{X}}({\varvec{s}},t)$ is the position of the solid wall in Lagrangian coordinates ${\varvec{s}}=({s}_{1},{s}_{2})$; ${\delta }_{ij}$ is the Kronecker delta; and ${{\varvec{B}}}_{L}$ is the Lagrangian force exerted on the wall by the fluid. The product $Eh$ and coefficient ${E}_{I}$ represent the stretching and bending stiffnesses, respectively. The matrices $\varphi$ and $\gamma$ represent in- and out-of-plane effects as a function of Poisson’s ratio $\widehat{\nu }$, respectively defined as

11 $$\begin{array}{c}\varphi =\left(\begin{array}{cc}1& \frac{1}{2\left(1+\widehat{\nu }\right)}\\ \frac{1}{2\left(1+\widehat{\nu }\right)}& 1\end{array}\right) , \gamma =\left(\begin{array}{cc}1& 1\\ 1& 1\end{array} \right).\end{array}$$

The compliant wall equations (Eqs. 10 and 11) are solved by a non-linear finite element method (FEM),^18^ where three-node triangular elements with six degrees-of-freedom are used to describe the solid deformation.^15^ A corotational scheme is used to handle the large displacements and small-strain deformations in the structural solver. Each discrete element obeys Kirchhoff–Love plate theory in their local coordinate system. Thus, the geometrical non-linearity is handled by coordinate transformation.^15,18^ A second-order-accurate iterative time-stepping strategy is employed in the solver. Further details of the FEM solver can be found elsewhere.^18^ In this work, the solid computational domain consists of $\sim \text{8000}$ triangular elements and $\sim 4000$ nodes, where the mesh size of the solid employed here satisfies the definition of comparability of Zhao *et al*.^50^ ($\Delta s\le 2\Delta x$) to ensure the stability of the fluid–solid coupling (described in what follows).

#### Immersed Boundary Method (IBM)

The fluid and solid domains are coupled *via* an immersed boundary method (IBM).^22^ IBM regulates the force term ${\varvec{b}}$ in Eq. 6 and enforces no-slip, no-penetration boundary conditions at the fluid–solid interface.^35^ The Lagrangian-interface force exerted on the wall by the fluid ${{\varvec{B}}}_{L}$ is calculated using a direct forcing scheme^22^ given by

12 $$\begin{array}{c}{{\varvec{B}}}_{L}=2\frac{\rho }{\Delta t}\left({{\varvec{V}}}_{f}\left({\varvec{s}},t\right)-{{\varvec{V}}}_{s}\left({\varvec{s}},t\right)\right),\end{array}$$

where ${{\varvec{V}}}_{s}\left({\varvec{s}},t\right)$ and ${{\varvec{V}}}_{f}\left({\varvec{s}},t\right)$ are the Lagrangian solid and fluid velocities at a material point ${\varvec{X}}\left({\varvec{s}},t\right)$ on the wall. ${{\varvec{V}}}_{f}$ is obtained from the Eulerian fluid velocity ${\varvec{v}}\left(x,t\right)$ by the expression

13 $$\begin{array}{c}{{\varvec{V}}}_{f}\left({\varvec{s}},t\right)=\int {{\varvec{v}}}^{\boldsymbol{*}}\left({\varvec{x}},t\right)\delta \left({\varvec{x}}-{\varvec{X}}\left({\varvec{s}},t\right)\right)d{\varvec{x}},\end{array}$$

where ${{\varvec{v}}}^{\boldsymbol{*}}=\frac{1}{\rho }\sum {e}_{i}{f}_{i}$ is an intermediate velocity. The corresponding body force ${\varvec{b}}$ in Eulerian coordinates can be calculated as

14 $$\begin{array}{c}b\left({\varvec{x}},t\right)=-\int {{\varvec{B}}}_{L}\left({\varvec{s}},t\right)\delta \left({\varvec{x}}-{\varvec{X}}\left({\varvec{s}},t\right)\right)d{\varvec{s}},\end{array}$$

where $\delta \left({\varvec{x}}-{\varvec{X}}\left({\varvec{s}},t\right)\right)$ is the Dirac delta function. The velocity is updated by ${\varvec{v}}={{\varvec{v}}}^{\boldsymbol{*}}+\frac{1}{2\rho } {\varvec{b}}\Delta t$. The multi-direct-forcing IBM enforces no-slip and no-penetration boundary conditions through an iterative procedure.^20^ Such a procedure can be summarized by the following steps, where steps one through five are iterated until the boundary conditions are satisfied:

- Step 0: $${{\varvec{v}}}^{*}=\frac{1}{\rho }\sum {e}_{i}{f}_{i}$$
- Step 1: $${{{\varvec{V}}}_{f}}^{n}=\int {{{\varvec{v}}}^{*}}^{,{\varvec{n}}}\delta d{\varvec{x}}$$
- Step 2: $${{{\varvec{B}}}_{L}}^{n}=2\frac{\rho }{\Delta t}\left({{{\varvec{V}}}_{f}}^{{\varvec{n}}}-{{\varvec{V}}}_{s}\right)$$
- Step 3: $${{\varvec{b}}}^{{\varvec{n}}}=-\int {{{\varvec{B}}}_{L}}^{n}\delta d{\varvec{s}}$$
- Step 4: $${{\varvec{v}}}^{{\varvec{n}}}={{{\varvec{v}}}^{*}}^{,{\varvec{n}}}+\frac{1}{2\rho } {{\varvec{b}}}^{{\varvec{n}}}\Delta t$$
- Step 5: $${{{\varvec{v}}}^{*}}^{,{\varvec{n}}+1}={{\varvec{v}}}^{{\varvec{n}}}$$

Upon completion, the corresponding Eulerian body and Lagrangian interaction forces at each new step are calculated explicitly *via* a summation of all intermediate values of such forces over all iterations, i.e., ${\varvec{b}}=\sum {{\varvec{b}}}^{{\varvec{n}}}$ and ${{\varvec{B}}}_{L}=\sum {{{\varvec{B}}}_{L}}^{n}$. These final forces are then substituted into their respective governing equations for the fluid (Eq. 6) and solid domains (Eq. 10). This iterative multi-direct-forcing IBM has been applied to a wide variety of FSI problems ranging from those related to the dynamics of inverted flexible plates^30^ to those concerning biological flow dynamics.^45,46^ A particular advantage of using this immersed-boundary lattice Boltzmann solver for FSI simulations lies in the ability of LBM to easily incorporate other physical effects as right-hand-side forcing terms of Eq. (1) (e.g., possibly non-Newtonian effects or the thermal considerations of this paper).

#### Catheter Model and Simulation Conditions

Inspired by IRRAflow® system, the considered catheter has a dual-lumen system separating injection and drainage ports to reduce likelihood of occlusions during operation. In our simulations, this is modeled as a rigid cylinder (of diameter 3.1 mm) with one drainage and six injection ports (all of diameter 1.5 mm) whose locations corresponds to those found in the IRRAflow® catheter. The tip is inserted 18 mm into the ventricular domain in order to ensure all ports are completely inside the ventricle.

We have employed the standard duty cycle of IRRAFlow® in all simulations: 20 s with three distinct time intervals corresponding to injection (1 s), observation (9 s), and drainage (10 s). The considered injection volume of ${Q}_{\text{inj}}=1\,\text{ ml}/\text{cycle}$ is often used in clinical practice.^37^ Ideally, the injected and drained volumes should be equal in order to prevent collapsing or possible bursting of the ventricle.^48^ The average velocity is calculated based on the injection volume, and spatially-uniform velocity profiles are prescribed at the injection and drainage sites during their corresponding operating time intervals. The onsets of these velocities are regularized-in-time by partition-of-unity-based functions to obtain appropriately smooth and physically-relevant simulations.

To the best of our knowledge, there is no prescribed injection temperature for this procedure, although it should be within a realistic range so as to avoid neural injury.^7,44^ The brain cells can tolerate low temperatures $(\sim 5\,{}\,{}^\circ\text{C} )$ for several weeks.^44^ However, they start to develop ischemia at temperatures that are slightly elevated above normal body temperature $(\sim 40\,{}^\circ\text{C} ).$^7^ Therefore, injections can only be performed at temperatures colder than the normal body temperature $(<37\,{}^\circ\text{C})$. In this work, we employ three injected fluid (medication) temperatures: 5, 20, and $37\,{}^\circ\text{C}$.

Fixed temperature boundary conditions and a bounce-back scheme are applied for the fluid flow simulations. The bounce-back method assures no-slip and no-penetration conditions by reversing the outgoing velocity distribution functions.^46^ This scheme is applied to the outer boundaries of the fluid domain as well as to the catheter boundaries (Figure S4 in SI), whereas the fixed temperature condition $(37\,{}^\circ\text{C} )$ is only enforced on the outer boundaries (i.e., of the rectangular prism) by Eq. (4).^31^

#### Non-dimensionalization of the Problem Parameters

Simulations are performed in non-dimensional lattice units, where the governing equations are non-dimensionalized by four reference parameters: a density $\rho$ (fluid density), a length $L$ (catheter diameter$L={D}_{\text{cath}})$, a time ${t}_{\text{ref}}$ (injection time ${{t}_{\text{ref}}=t}_{\text{inj}}=1$ s), and a temperature difference $\Delta T={T}_{\text{inj}}-{T}_{\infty }$ (the temperature between the injected fluid and the patient’s body). This yields corresponding non-dimensional quantities describing fluid flow: Strouhal number ${\text{St}}= L/(U{t}_{\text{ref}})$, Reynolds number ${\text{R}}{\text{e}}=\rho UL/\mu$, Froude number ${\text{Fr}}=U/\sqrt{gL}$, Prandtl number ${\text{Pr}}=\mu /\rho \alpha$, Rayleigh number ${\text{Ra}}=\rho {\upbeta \Delta }{TL}^{3}g/\mu \alpha$, and flow velocity (in lattice units) of $U={Q}_{\text{inj}}/{\sum A}_{\text{inj}}$ (where ${\sum A}_{\text{inj}}$ represents the total area of the injection ports). The non-dimensional quantities describing the solid wall are given by bending coefficient ${K}_{B}= {E}_{I}/(\rho {U}^{2}{L}^{3})$, tension coefficient ${K}_{s}= Eh/(\rho {U}^{2}L)$, and mass ratio $M= {\rho }_{s}h/(\rho L)$. The ventricle wall material parameters are assumed to be same as the surrounding white matter and nearly incompressible (i.e., $E=1.88 \,\text{kPa}$, $\widehat{\nu }=0.43$),^11^ with a thickness corresponding to $h=2{D}_{\text{cath}}=6.2 \,\text{mm}$. Hence the flexural rigidity, bending, and tension coefficients can be calculated as ${E}_{I}=4.57\times {10}^{-5}\,\text{ Pa }{\text{m}}^{3}$,${ K}_{B}=1.56\times {10}^{2}$, and ${K}_{s}=3.83\times {10}^{2}$, respectively. The wall density is considered to be the same as the fluid density, yielding a mass ratio $M=2$.

#### Numerical Simulations

All simulations are performed using a uniform mesh with a lattice spacing of $L/\Delta x=10$ in all directions ($\sim 3.75\times {10}^{6}$ fluid nodes) and a timestep of $\Delta t=3.95\times {10}^{-4}{\text{s}}$. The chosen timestep satisfies both the stability condition of the FSI solver as well as the CFL constraint of the advection–diffusion algorithm and, further, is insensitive to changes in other simulation parameters. Mesh independency studies have been performed to ensure convergence of the numerical results for the parameters employed in this work. The fluid in the ventricle is modeled to have a dynamic viscosity of $\mu =7\,\text{mPa s}$ with thermodynamic and transport properties of saline water.^32^ For the case of an injection temperature of $5\,{}^\circ\text{C}$, this yields peak Reynolds and Rayleigh numbers of ${\text{Re}}=46$ and ${\text{Ra}}=2204$, respectively.

### Advection–Diffusion Solver for Dye Simulations

The evolution of a dye concentration $c({\varvec{x}},t)$ subjected to a velocity field ${\varvec{v}}({\varvec{x}},t)$ can be governed by the homogeneous advection–diffusion equation,^4^ i.e.,

15 $$\begin{array}{c}\frac{\partial c}{\partial t}+{\varvec{v}}\cdot \nabla c-D{\nabla }^{2}c=0,\,t>{t}_{0},\end{array}$$

where $D$ is the diffusion coefficient ($D=1.5\times {10}^{-9}{\text{m}}^{2}/{\text{s}}$)^32^ and where ${t}_{0}$ is the time corresponding to the start of dye injection. Null conditions are utilized before injection (i.e., $c=0, t<{t}_{0}$). The velocity field ${\varvec{v}}$ can be provided by any suitably smooth data; in the present study, such data is produced numerically through our lattice Boltzmann-based FSI solver described above. Dye injection along an inlet boundary $\partial {\Omega }_{inlet}$ over a time interval $t\in [{t}_{1}, {t}_{2}]$ corresponds to a Dirichlet boundary condition given by

16 $$\begin{array}{c}c\left({\varvec{x}},t\right)=1,\, x\in \partial {\Omega }_{\text{inlet}}, \,t\in \left[{t}_{1},{t}_{2}\right].\end{array}$$

At all other boundaries, including rigid walls (where, ostensibly, ${\varvec{v}}=0$), a zero-flux (homogeneous Neumann) boundary condition is applied at all-time instances and is given by

17 $$\begin{array}{c}\nabla c\left({\varvec{x}},t\right)=0,\, x\in \frac{\partial {\Omega }_{\text{inlet}}}{\partial\Omega },\, t>{t}_{\text{o}}.\end{array}$$

Zero flux conditions are also applied at the outlet, enabling the dye to leave the domain without reflection.^4^ We note here that Eqs. (16) and (17) are not explicitly applied to the ventricle wall, as it is not an explicit boundary in the computational domain of the advection–diffusion model (whose boundaries are only the rigid walls, i.e., the outer prism and the catheter). The subsequent solver takes, as input, the flow fields produced by our FSI solver (which, indeed, respect the elastic boundary of the ventricle, and are not-penetrating).

#### Numerical Methodology

Solution of Eqs. (15–17) is facilitated by a recently-introduced solver^4^ based on a Fourier continuation (FC) methodology^1–3,5^ for accurate Fourier series expansions of non-periodic functions. For a dimension ${x}^{j}$ defined on the unit interval $[0, 1]$ and uniformly discretized by $N$ points (i.e., ${x}_{n}^{j}=n/(N-1), n=0,\dots , N-1$), the FC algorithm appends a small number of discrete points to the original pointwise function values $c\left({x}_{n}^{j}\right)=c\left({x}_{n}^{j}, t\right)$ in order to form a trigonometric polynomial ${c}_{\text{cont}}\left({x}^{j}\right)$ that is periodic on an interval of length $l>1$. Such an interpolating polynomial can be represented by a band-limited Fourier series that is given by

18 $$\begin{array}{c}{c}_{\text{cont}}\left({x}^{j}\right)=\sum\limits_{k=-M}^{M}{A}_{k}{e}^{\frac{2\pi ik{x}^{j}}{l}},\end{array}$$

and that agrees well with the original values $c\left({x}_{n}^{j}\right)$, i.e., ${c}_{\text{cont}}\left({x}^{j}\right)=c\left({x}_{n}^{j}\right)$. Corresponding spatial derivatives of Eqs. (15–17) can then be computed by exact term-wise differentiation of Eq. (18), yielding

19 $$\begin{array}{c}\frac{\partial c}{\partial {x}^{j}}\left({x}_{n}^{j}\right)=\frac{\partial {c}_{\text{cont}}}{\partial {x}^{j}}\left({x}_{n}^{j}\right)=\sum\limits_{k=-M}^{M}\left(\frac{2\pi ik}{l}\right){A}_{k}{e}^{\frac{2\pi ik{x}_{n}^{j}}{l}}.\end{array}$$

The resulting function ${c}_{\text{cont}}$ can be interpreted as a discrete representation of a smooth function that is periodic on a slightly larger interval and that can hence be approximated to high order by a Fourier series representation (whose coefficients ${A}_{k}$ in Eq. (19) can be calculated efficiently by a Fast Fourier Transform). Further details on the construction of Fourier continuations, including for Neumann boundary conditions, can be found elsewhere.^1–4^

A high-order explicit time integration scheme is utilized to complete the numerical treatment of the advection–diffusion system.^4^ Such time marching incurs linearly-scaling Courant-Friedrichs-Lewy (CFL) constraints on the timestep.^2,3,5^ Together with the FC-based spatial scheme, the overall solver enables high-order accuracy and a faithful preservation of the dispersion/diffusion characteristics of the underlying continuous operator (i.e., no ``pollution" errors that compound over space and time^2–4^). The latter property is particularly important for avoiding non-physical (i.e., numerically-induced) diffusion in simulated dye concentrations.

### Fluid Dynamics-Transport Analysis

Results obtained from the FSI and advection–diffusion solvers are quantified using three-dimensional fluid blending due to the injection and dye distribution. The average vorticity magnitude inside the ventricle is used as a metric to quantify the overall *fluid blending* in the ventricle using Eq. (20), i.e.,

20 $$\frac{1}{{V_{{{\text{vent}}}} }}\iiint_{{V_{{{\text{vent}}}} }} {\left\| {\nabla \times \user2{v}} \right\|_{2} dV}$$

where ${V}_{\text{vent}}$ denotes the volume of the brain ventricle prior to injection $({V}_{\text{vent}}=17.4\, {\text{ml}})$.

The delivery performance is quantified as the occupied volume at the end of the catheter duty cycle with different dye concentrations thresholds $({c}_{0})$:

21 $$\frac{1}{{V_{{{\text{vent}}}} }}\iiint\nolimits_{{V_{{{\text{vent}}}} }} {\left\{ {\begin{array}{*{20}l} {1,} \hfill & {c\left( {{\varvec{x}},t = 20} \right) > c_{0} } \hfill \\ {0,} \hfill & {otherwise} \hfill \\ \end{array} } \right.}dV.$$

Ultimately, Eq. (20) serves as a metric to quantify flow blending due to injection, and Eq. (21), together with the overall dye concentrations, serves as a metric to quantify the overall “mixing” of the injected dye (i.e., how much of the dye remains within the volume after drainage).

### Summary of the Modeling Framework

**FIGURE 2:**
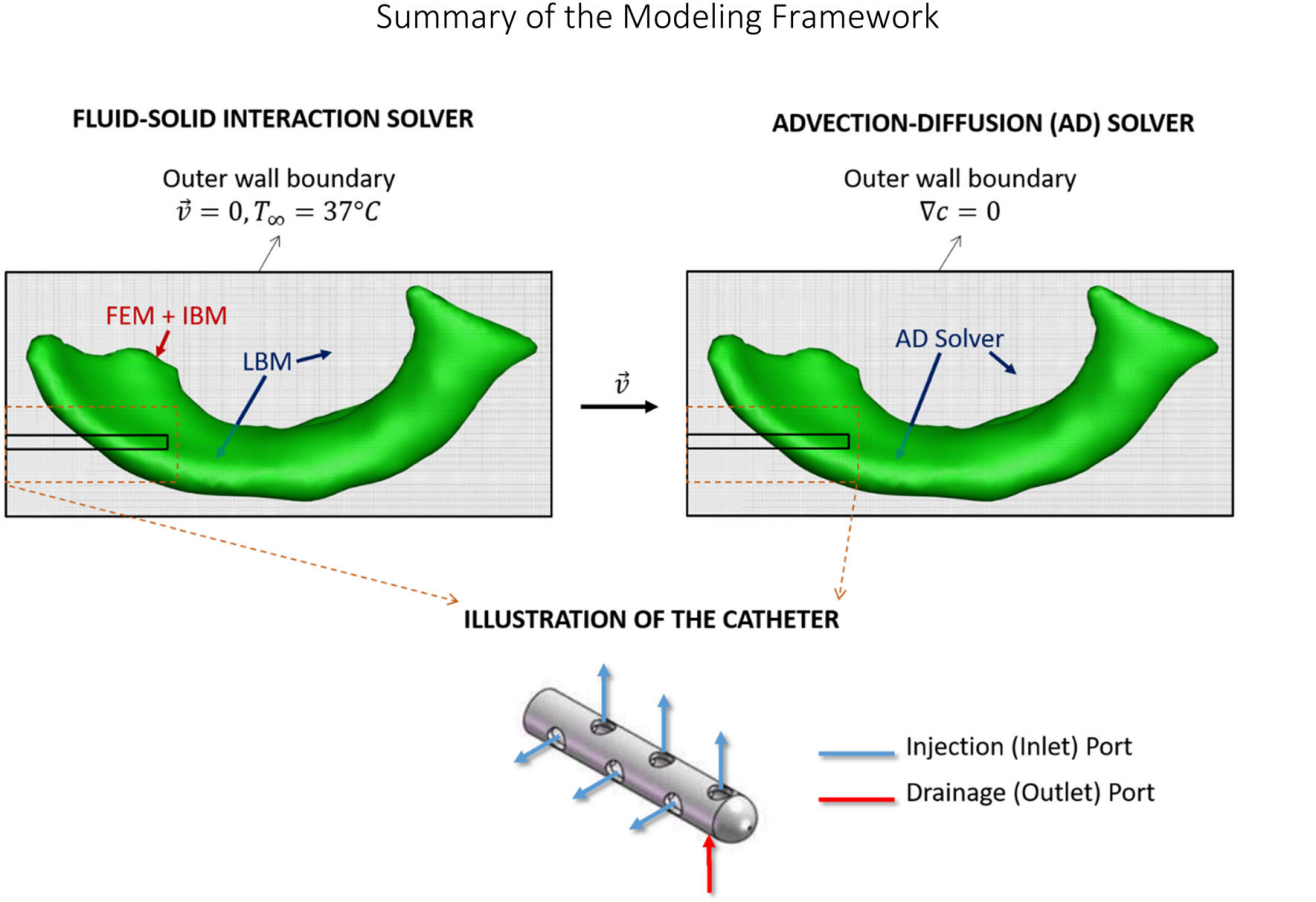
A summary of the overall modeling framework: the FSI solver, the advection–diffusion solver, and an illustrative schematic of the catheter model with its corresponding injection and drainage ports.

Figure 2 presents a monolithic diagram of the overall numerical methodology and boundary conditions (the FSI solver, the advection–diffusion solver, and the catheter configuration). The thermal flow equations are solved for every fluid mesh element (see Fig. S4a). The finite element method is only applied to the cerebral ventricle geometry. The immersed boundary method is applied to the near vicinity of the solid geometry to couple the solid and fluid domains. The velocity vectors obtained by our FSI solver are used as input for our advection–diffusion (AD) solver, which then solves the AD equation on every fluid mesh element. The inlets and the outlet defined for the problem are located on the modeled catheter.

## Results

### Velocity Field Results

The results presented in this subsection have been obtained with the LBM-based FSI solver described above. Simulations are performed for three injection temperatures and two different patient positions: supine (lying on the back with the face upwards) and prone (lying on the stomach with the face downwards). Other body positions are not considered since they have been demonstrated to be clinically impractical.^34^ The injection procedures are performed at cold ($5\,{}^\circ\text{C}$), room ($20\,{}^\circ\text{C}$), and body ($37\,{}^\circ\text{C}$) temperatures, where the lattermost represents the “baseline” performance of the catheter (which is independent of patient posture since buoyancy force is zero *via* Eq. (5), see Supplementary Information).

**FIGURE 3:**
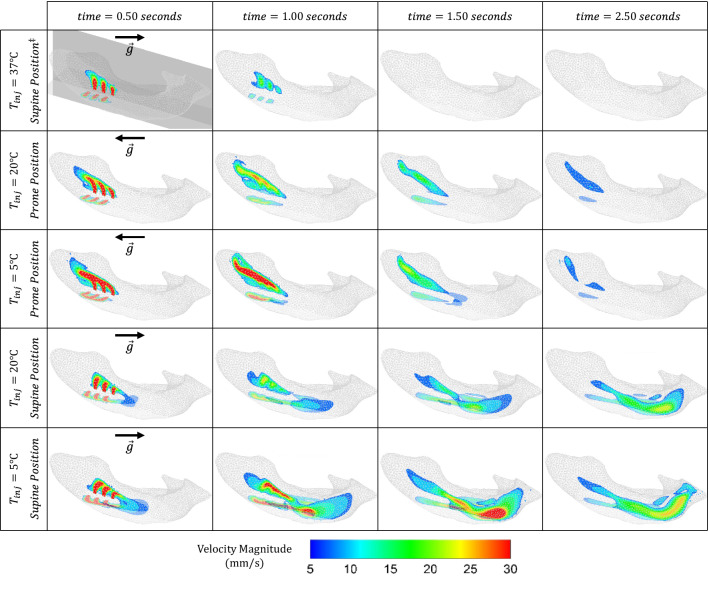
Velocity fields at two cross-sections in the ventricle at various snapshots in time (columns) for different injection temperatures and patient positions (rows). The second column (time $t=1$ s) corresponds to the end of the injection period. $^{\ddagger }$ Note that for injections at body temperature ($37\,{\,{}^\circ\text{C} }$), the solution is independent of patient posture since buoyancy force is zero *via* Eq. (5) and hence only one position has been provided.

**FIGURE 4:**
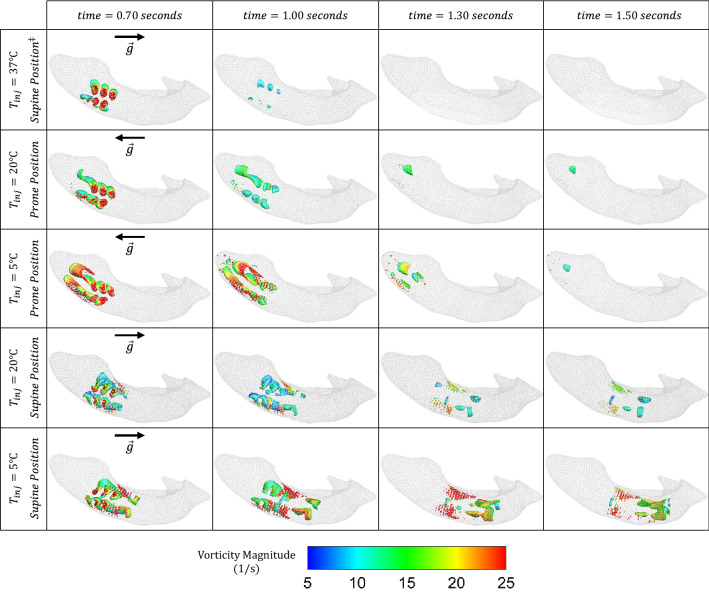
Vortex fields (generated using the Q-criterion method with $Q=0.1\, {\text{s}}^{-1}$) in the ventricle at various snapshots in time (columns) for different injection temperatures and patient positions (rows). The second column (${\text{time}}=1$ s) corresponds to the end of the injection period. $^{\ddagger }$ Note that for injections at body temperature ($37\,{\,{}^\circ\text{C} }$), the solution is independent of patient posture (supine/prone) since buoyancy force is zero *via* Eq. (5) and hence only supine position has been provided.

**FIGURE 5:**
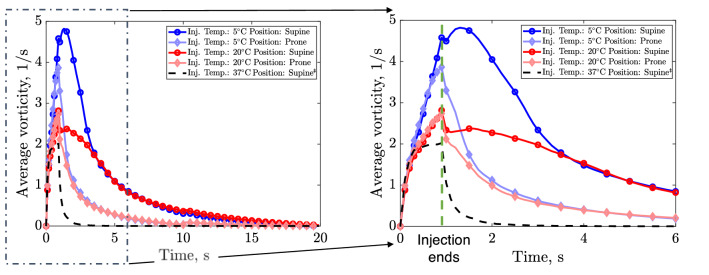
(Left) Average vorticity inside the brain ventricle for each case during the complete catheter cycle (up to ${\text{time}}=20$ s). (Right) A zoom of the same values during the first 6 s (where the end of injection is indicated). $^{\ddagger }$ Note that for injections at body temperature ($37\,{\,{}^\circ\text{C} }$), the solution is independent of patient posture (supine/prone) since buoyancy force is zero *via* Eq. (5) and hence only supine position has been provided.

Simulated snapshots, at various times, of the velocity and vorticity fields inside the ventricle are presented in Figs. 3 and 4, respectively. Table 1 additionally presents the flow die-out time (corresponding to the time at which maximum velocity drops below 0.5 mm/s) for the three injection temperatures and two patient positions. As a metric for quantifying the three-dimensional fluid blending due to the injection (Eq. 20), Fig. 5 presents the average vorticity for each configuration.

**TABLE 1:** Flow die-out time (the time after which maximum velocity drops below 0.5 mm/s) corresponding to each case presented in Fig. 3.

Injection condition	Flow die-out time
$37\,{}^\circ\text{C}$- Supine^a^	1.3 s
$20\,{}^\circ\text{C}$- Prone	3.3 s
$5\,{}^\circ\text{C}$- Prone	3.5 s
$20\,{}^\circ\text{C}$- Supine	9.4 s
$5\,{}^\circ\text{C}$- Supine	8.8 s

^a^Note that for injections at body temperature ($37\,{\,{}^\circ\text{C} }$), the solution is independent of patient posture (supine/prone) since buoyancy force is zero *via* Eq. (5) and hence only supine position has been provided

Every velocity magnitude image in Fig. 3 displays two overlaid cross-sections (opaque and transparent) intersecting at the radial centerline of the catheter, where the first row corresponds to the $37\,{}^\circ\text{C}$ case. With no (expected) thermal effect at this baseline, the velocity vectors are damped out and disappear at the end of injection ($t\approx 1.3$ s). When a temperature difference is introduced (second through fifth rows), one can observe significant changes in the velocity field compared to the baseline for both prone and supine positions. For the former injected at non-body temperatures, we observe a non-negligible fluid velocity in the occipital horn (see Fig. 1a) of the ventricle and hence persistent clot irrigation until a die-out time of 8.8–9.4 s (Table 1). A non-negligible velocity can be observed at the prone position, tending instead towards the anterior horn (see Fig. 1a) and lasting (actively irrigating) for roughly a third of the time, i.e., 3.3–3.5 s. For both cold ($5\,{}^\circ\text{C}$) and room ($20\,{}^\circ\text{C}$) temperatures, the velocity distributions (Fig. 3) as well as the die-out times (Table 1) are comparable. However, velocity magnitudes at $5\,{}^\circ\text{C}$ reach values twice as large as those from injecting at $20\,{}^\circ\text{C}$. Videos corresponding to the time evolution of all cases in Fig. 3 are provided in Supplementary Information.

Snapshots of the time evolution of the vortex structures corresponding to each configuration is presented in Fig. 4. The vortices have been visualized using the Q-criterion method with iso-surfaces at $Q=0.1 \ {\text{s}}^{-1}.$ The injected fluid introduces vortices in all cases. At baseline, these vortices separate from the catheter after the end of injection, disappearing quickly due to the smaller die-out time. When thermal effects are introduced, the vortices bend in directions based on patient position. At supine, we observe some vortices pinching off from the initial vortex cores to form additional vortices, ultimately traveling towards the occipital horn as expected from the velocity fields of Fig. 3. Similarly, prone, we observe vortex cores traveling towards the anterior horn. However, these structures remain intact (i.e., no pinch-off), and thus their sizes are larger than those at supine. Injection at temperatures colder than baseline results in stronger vortices (in terms of magnitude) occupying larger regions.

Figure 5 provides a quantitative assessment of the *fluid blending* caused by the injected fluid *via* the space-averaged vorticity for each configuration. We observe that vorticity curves produced by all injections are similar at their onset. However, midway through each injection, the subsequent evolution of the average vorticity begins to differ drastically as a function of temperature. We note that the coldest injection results in up to twice as much blending than at room temperature. Following the end of injection (at $t\approx 2 \ \text{s}$ and $t\approx 4 \ \text{s}$ for prone and supine, respectively), the differences between the cold and room temperature cases start to diminish, and the average vorticity becomes similar for both positions well before the onset of drainage. Although such thermal effects on vorticity disappear before drainage ($t=10 \ \text{s}$), we observe that postural effects continue to persist until the end of the complete catheter cycle ($t=20$ s).

### Evolution of Injected Fluids

For each case of temperature and position, one can expect a unique dye evolution/delivery due to the differences in velocity field patterns and vortical structures (Figs. 3 and 4). In order to investigate the distribution of the injected dye, these velocity vectors are then employed in the FC-based advection–diffusion solver.

**FIGURE 6:**
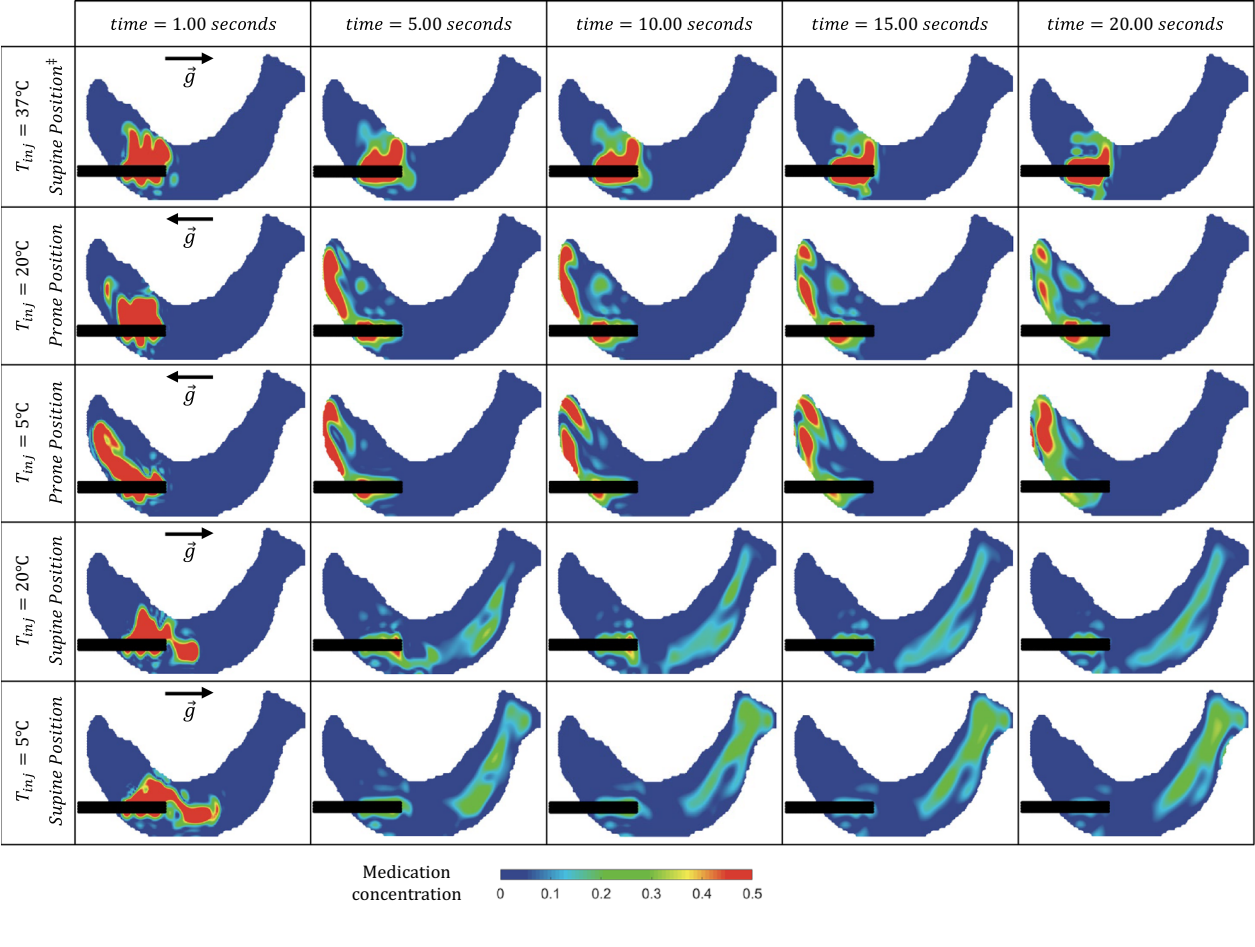
Simulated dye concentrations on a plane (slice) of the ventricle at various snapshots in time (columns) for different injection temperatures and patient positions (rows). The first (${\text{time}}=1.0 \ \text{s}$), third (${\text{time}}=10.0 \ \text{s}$) and fifth columns (${\text{time}}=20.0 \ \text{s}$) correspond to the end of the injection period, the beginning of drainage, and the end of the duty cycle (simulation), respectively. $^{ \ddagger }$ Note that for injections at body temperature ($37\,{\,{}^\circ\text{C} }$), the solution is independent of patient posture (supine/prone) since buoyancy force is zero *via* Eq. (5) and hence only supine position has been provided.

Figure 6 presents, for each configuration, the resulting simulated dye (e.g., medication) distributions at five instances, including at the end of injection ($t=1.0$ s), the start of drainage ($t=10.0$ s) and the end of the operating cycle ($t=20.0$ s).

Table 2 presents the corresponding “injected fluid utilization rate” calculated as the ratio of final remaining dye volume to the original injected volume. For injection at baseline (first row), we observe that the dye diffuses only within the near vicinity of the catheter, and a large portion of the medication ($35.5\%$) is drained back by the catheter without having been utilized (i.e., not having reached other parts of the ventricle). For other cases, we observe significant effects on the delivery patterns due to thermal and postural conditions. These buoyant effects (Eq. 5) enable medication to be delivered to a larger volume than at baseline. At supine position, for example, dye travels towards the occipital horn of the ventricle and, at prone, towards the anterior horn. Since the catheter is inserted into the latter, a larger amount of dye is drained back at prone than at supine. Additionally, injection at the coldest temperature results in higher concentrations near the ventricle walls when compared with room temperature. Subsequently, a smaller volume of dye is drained during the drainage of the catheter, and the medication utilization is higher (more remains post-drainage).

**TABLE 2:** Medication utilization rate: ratio (as a percentage) of final remaining dye volume to the original injected volume.

Injection condition	Remaining dye volume, %
$37\,{}^\circ\text{C}$- Supine^a^	64.5
$20\,{}^\circ\text{C}$- Prone	76.2
$5\,{}^\circ\text{C}$- Prone	81.3
$20\,{}^\circ\text{C}$- Supine	89.2
$5\,{}^\circ\text{C}$- Supine	97.1

^a^Note that for injections at body temperature ($37\,{\,{}^\circ\text{C} }$), the solution is independent of patient posture (supine/prone) since buoyancy force is zero *via* Eq. (5) and hence only supine position has been provided

**FIGURE 7:**
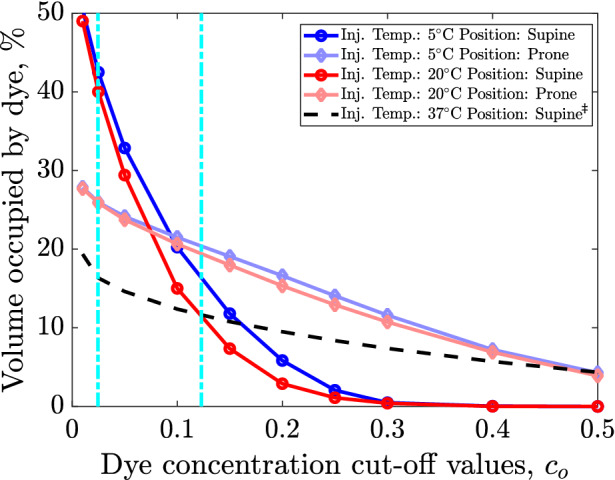
Percentage of total volume occupied after drainage at different threshold values $${c}_{0}$$ of dye/medication concentrations (e.g., the volume occupied by $$c\left(x\right)>{c}_{o}$$). The dashed lines demarcate the lower and upper limits of optimally-effective dye (e.g., drug) concentrations.^8^ $$^{ \ddagger }$$ Note that for injections at body temperature ($$37\,{\,{}^\circ\text{C} }$$), the solution is independent of patient posture (supine/prone) since buoyancy force is zero *via* Eq. (5) and hence only supine position has been provided.

Figure 7 presents the percentage of total volume occupied at the end of the catheter duty cycle at different threshold values ${c}_{0}$ of dye concentrations (cf. Eq. 21). The optimum effectiveness of tPA is achieved at concentrations between $5$ and $25$ nM (routine clinical treatment procedure for IVH involves the addition of $\approx 2 \text{mg}$ tPA per $500$ ml saline water),^8^ corresponding to concentrations of $2.46$ and $12.3\%$ in our simulations (vertical dashed lines in Fig. 7). The injected fluid distribution performance can be quantified as the volume occupied by the dye that is within the optimally-effective concentration range, presented in Table 3 as a percentage of total ventricle volume.

**TABLE 3:** Final percentage of the ventricle occupied by dye that is within the optimally-effective concentration range (dashed lines of Fig. 7).

Injection condition	Volume of ventricle with dye in optimum range, %
$37\,{}^\circ\text{C}$- Supine^a^	15.3
$20\,{}^\circ\text{C}$- Prone	23.8
$5\,{}^\circ\text{C}$- Prone	25.2
$20\,{}^\circ\text{C}$- Supine	35.1
$5\,{}^\circ\text{C}$- Supine	38.4

^a^Note that for injections at body temperature ($37\,{}^\circ\text{C}$), the solution is independent of patient posture (supine/prone) since buoyancy force is zero *via* Eq. (5) and hence only supine position has been provided

We observe in Fig. 7 that the lowest diffusion performance is for injection at baseline, where only $15.3\%$ of the ventricle contains optimum concentration levels of dye. Thermal effects appear to increase both this diffusion as well as the *effective dye volume*. However, we note that patient position appears to have a greater effect than injection temperature. The supine position results in more volume covered with optimal concentrations than at prone. Consistent with Fig. 6, delivery appears limited to a small region at prone, and $5\%$ of the ventricle has more than $50\%$ concentration. At supine, however, medication diffuses through a much larger volume and the maximum final concentration in the ventricle is less than $30\%.$ Additionally, injection at $5\,{}^\circ\text{C}$ increases the effective dye volume slightly when compared to $20\,{}^\circ\text{C}$ (by 3.3 and 1.4% for supine and prone, respectively).

## Discussion

This work studies the thermal and postural effects on irrigation patterns and injected fluid mixing of a newly-developed self-irrigating catheter to utilize an active fluid exchange mechanism for treating intraventricular hemorrhages. Through robust numerical simulations, we have investigated clot irrigation potential (*via* consideration of velocity fields) and drug delivery performance (*via* consideration of vortex structures and dye evolution) under different injection temperatures and patient positions.

Overall, we observe that injection at baseline ($T={T}_{\infty }=$ 37$\,{}^\circ\text{C}$) yields the least favorable outcome for treatment. Fluid flow and subsequent dye/medication diffusion remains restricted to regions near the original injection sites (first row of Figs. 3 and 4). Although this is to be expected from the lack of corresponding buoyancy effects (force density is zero in Eq. 5), such limited movement implies poor clot irrigation and an ineffective distribution of injected dye/medication (which does not reach much of the ventricle). Additionally, more of the injected dye is drained (Table 2), and what little remains is generally at concentrations outside of the optimally-effective range (Fig. 7). This implies that much of any injected medication would go underutilized.^8^

On the other hand, buoyant effects at temperatures $T<37 \,{}^\circ\text{C}$ clearly provide significant enhancements. A comparison of velocity fields in Fig. 3 demonstrates a drastic change in irrigation patterns at both 5$\,{}^\circ\text{C}$ and 20$\,{}^\circ\text{C}$, regardless of patient position. Indeed, the fluid preserves significant energy and momentum up to seven times longer than at baseline (Table 1), and vortex structures occupy larger volumes of the ventricle (Figs. 4 and 5). Vorticities also persist beyond the die-out time due to the velocity thresholds employed. Overall, this implies that injection at any temperature colder than body will yield a larger volume of undrained medication (Table 2) spread over a larger region for a longer duration, ultimately improving the treatment potential of such a procedure.

As can be further observed in Figs. 4, 5 and 7, such thermal effects strengthen as temperature decreases: 5$\,{}^\circ\text{C}$ yields even larger dye volumes and more persistent irrigation patterns than 20$\,{}^\circ\text{C}$. Indeed, Eq. (5) implies that the larger the temperature difference (from baseline), the larger the expected buoyancy forces. At 5$\,{}^\circ\text{C}$, the dye snapshots of Fig. 6 demonstrate diffusion and dissipation that result in more clinically-favorable^8^ (between $2.5\%$ and $12.3\%$) concentration levels. With such optimum concentrations covering larger portions of the ventricle, less of an injected medication remains underutilized.

Different patient positions also yield very different irrigation patterns and fluid mixing (except at baseline, cf. Eq. 5). In clinical applications, targeting the occipital horn (a more complex geometry than the anterior horn) might be preferred since it is a common location of persistent IVH post-treatment clots.^36,41^ To this end, our results clearly suggest positioning the patient face-up (supine) instead of face-down (prone) for better irrigation (Figs. 3 and 4) and drug delivery (Fig. 6). Indeed, the significantly higher overall die-out time (Table 1), remaining dye volume (Table 2), and amount of optimum dye concentration (Table 3) implies more efficient drug utilization and longer hematoma irrigation at supine for both 5$\,{}^\circ\text{C}$ and 20$\,{}^\circ\text{C}$.

We note here that we have used a healthy, generalized, anatomically-realistic brain ventricle; a more accurate model might contain patient-specific characteristics including ventricle size and alignment as well as irregularly-shaped features. We have also fixed the catheter location and orientation in order to fully isolate and study thermal/postural effects. Although there may also be some differences in insertion angle and orientation in clinical practice, our catheter model is based on current manufacturer recommendations. Future work entails investigating the effects of different lateral ventricle morphologies and catheter placement (can be provided by post-operation tomography images). Additionally, we have not considered chemical reaction between injected tPA solution and hematomas in our simulations. Our results only elucidate the path of diffusion and not the actual activated interaction between tPA and hematomas (where, with a finite reaction rate, medication can be consumed during clot breaking). The addition of such a reaction term to the governing model (Eq. 15) is a subject of our future work.

Some additional limitations should be mentioned. To the best of our knowledge, the properties of an intraventricular hematoma (e.g., transport and thermal) are not well established and there is a dearth of literature. As such, multi-phase fluid models are not feasible. Hence, we have employed a single-phase model by assuming the hematoma is a uniform mixture of blood and cerebrospinal fluid, where medication immediately mixes with the hematoma at the injection port. Further research into the properties of a hematoma may one day enable consideration of fluid–fluid interactions; however, conclusions drawn from our study would still be reasonably accurate since increased thermal forces (lower injection temperatures) enhance irrigation and drug distribution in any case.

Overall, this work demonstrates that both temperature and patient position are important factors in catheter efficacy. Results suggest that treatment administered at a high temperature difference (e.g., $5\,{}^\circ\text{C}$) increases efficiency and that irrigation can be directed as necessary *via* patient positioning. Our unique modeling approach may help facilitate rapid assessment of drug delivery performance before treatment. Indeed, the first two days are most critical for IVH patients, and this work has been motivated by the overarching goal of faithfully producing treatment-specific simulations towards enabling *a priori* catheter performance estimation within a few hours.

## Supplementary Information

Below is the link to the electronic supplementary material.

Supplementary file1 (PDF 705 kb)

Supplementary file2 (MP4 642 kb)

Supplementary file3 (MP4 642 kb)

Supplementary file4 (MP4 1620 kb)

Supplementary file5 (MP4 1350 kb)

Supplementary file6 (MP4 1946 kb)

Supplementary file7 (MP4 1418 kb)

Supplementary file8 (MP4 2563 kb)
