# Supplementary material for: Thermal and Postural Effects on Fluid Mixing and Irrigation Patterns for Intraventricular Hemorrhage Treatment
Source: Ann Biomed Eng. 2023 Jan 21;51(6):1270–83. doi: 10.1007/s10439-022-03130-9 (PMC10172237; doi:10.1007/s10439-022-03130-9)
Supplement: Supplementary file 1 — Supplementary file1 (PDF 705 kb) [file 10439_2022_3130_MOESM1_ESM.pdf]

SUPPLEMENTARY INFORMATION

Validation of Thermal Lattice-Boltzman Solver

The numerical solver uses the Boussinesq approximation for thermal fluid flow problems. We used the Rayleigh-Benard convection benchmark problem provided by de Vahl Davis [1] to show the accuracy of our solver. The geometry and the boundary conditions used by de Vahl Davis [1] are given in Figure S1.a. In order to show the accuracy of our solver in a three-dimensional problem, we used the geometry of a cube and then applied insulation (no-flux) boundary conditions to the *extra* walls in z-direction (Figure S1.b). Additionally, we changed the problem orientation to Eulerian *x* and *y* directions, and obtained identical results for all cases. Figure S2 shows the contours of temperature, horizontal/vertical velocity, and the velocity vector field at a slice that passes through the center of the cube. The simulations are performed for a Prandtl number of 0.71 and Rayleigh numbers of 10^3 and 10^5. The results in Figure S2 are in excellent agreement with the results of de Vahl Davis [1].

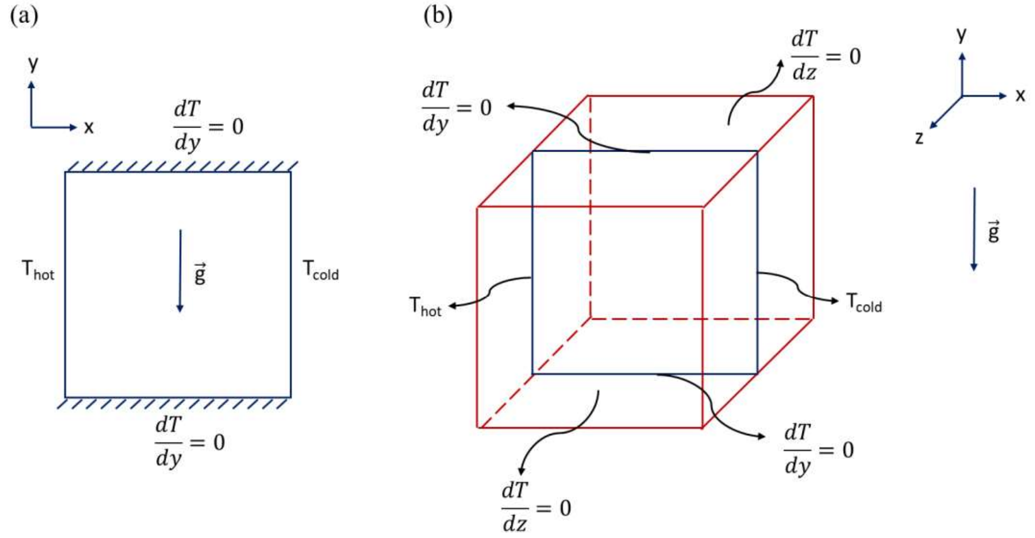

Figure S1. (a) Benchmark geometry used by de Vahl Davis [1], (b) cube geometry used for validating the three-dimensional solver.

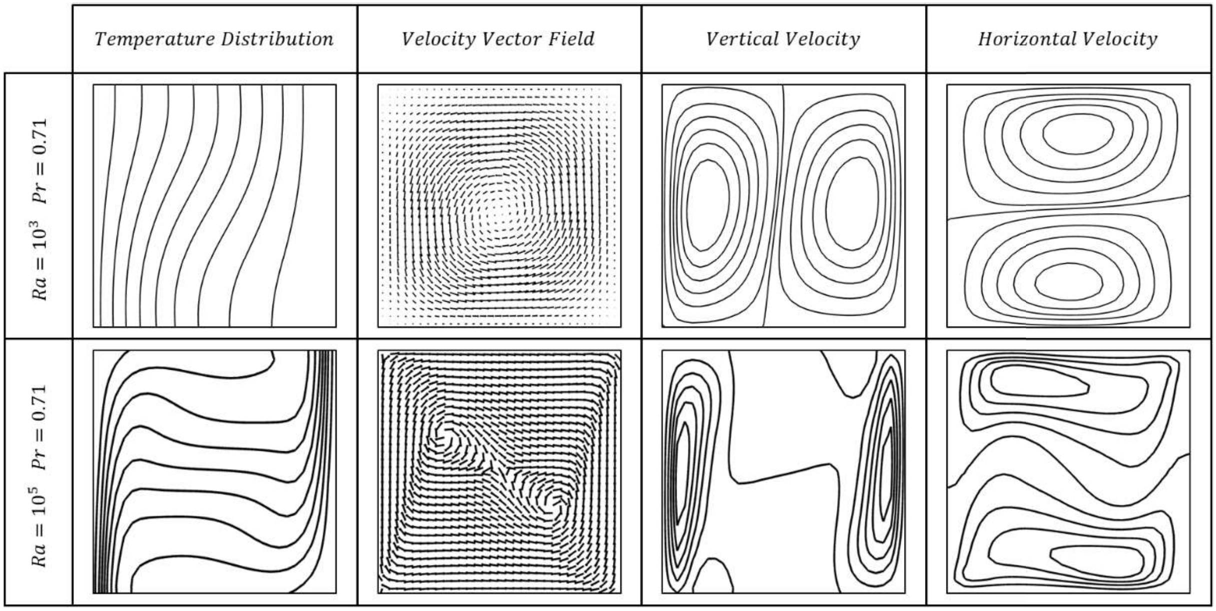

Figure S2. Results produced by the three-dimensional numerical solver at the central slice of the cube.

Validation of Fluid-Solid Interaction Solver

We used a three-dimensional flapping flag subjected to uniform flow [2] as a benchmark problem in order to test our fluid-solid interaction (FSI) solver. Figure S3.a shows an illustration of the problem geometry. The simulation is performed for a square flag with the Reynolds number of 100, stretching coefficient (K_s) of 1000 and bending coefficient (K_b) of 0.0001. Figures S3.b and S3.c present the transverse displacements of the points A and B, respectively. Our results show good agreement with the results of Huang and Sung [2]. Our solver has been additionally validated on other configurations [3-5].

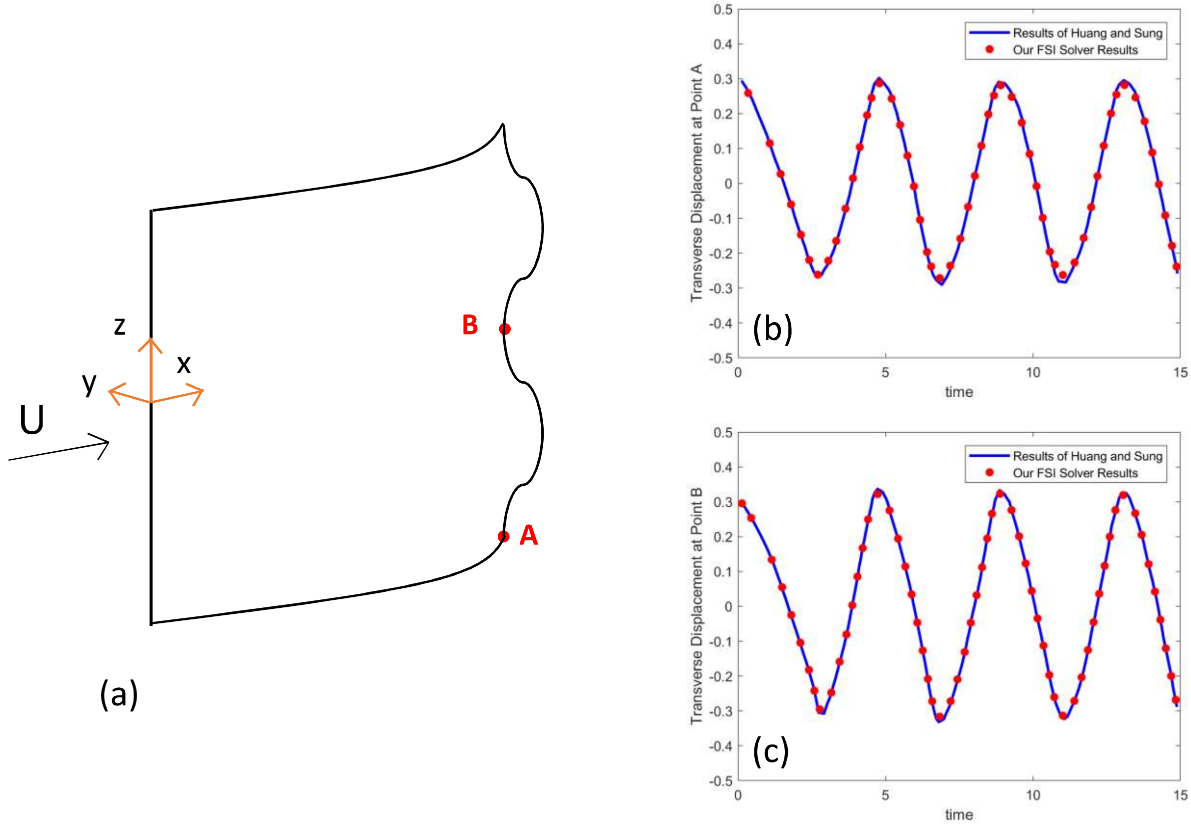

Figure S3. Benchmark problem for our FSI solver. (a) Illustration of the problem geometry, and (b,c) transverse displacements for point A and B, respectively.

Numerical Model

Figures S4.a and S4.b show the overall fluid mesh and the cerebral ventricle geometry. The boundary conditions of the thermal flow solver are applied to the boundaries of the mesh presented in Figure S4.a. The no-slip and no-penetration boundary conditions at the cerebral ventricle walls are enforced by the immersed boundary method (See Methods of main manuscript for details).

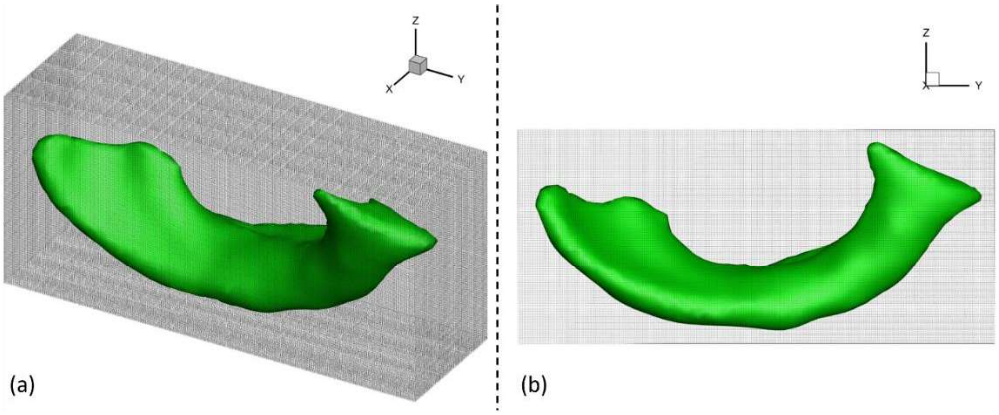

Figure S4. Fluid mesh and the cerebral ventricle geometry used in the simulations shown from (a) an isometric view, and (b) a front-view (x-plane).

References

[1] G. de Vahl Davis, "Natural convection of air in a square cavity: a bench mark numerical solution," *Int. J. Numer. Methods Fluids,* vol. 3, no. 3, pp. 249-264, 1983.

[2] W.-X. Huang and H. J. J. J. o. F. M. Sung, "Three-dimensional simulation of a flapping flag in a uniform flow," vol. 653, pp. 301-336, 2010.

[3] H. Wei, C. S. Herrington, J. D. Cleveland, V. A. Starnes, and N. M. Pahlevan, "Hemodynamically efficient artificial right atrium design for univentricular heart patients," *Physical Review Fluids,* vol. 6, no. 12, 2021-12-16 2021, doi: 10.1103/physrevfluids.6.123103.

[4] H. Huang, H. Wei, and X.-Y. Lu, "Coupling performance of tandem flexible inverted flags in a uniform flow," *Journal of Fluid Mechanics,* vol. 837, pp. 461-476, 2018.

[5] H. Wei, F. Amlani, and N. M. Pahlevan, "Direct 0D-3D coupling of a lattice Boltzmann methodology for fluid-structure hemodynamics simulations," *arXiv preprint arXiv:2112.05258,* 2021.

Attached Videos

Video1. Velocity fields at two cross-sections in the ventricle at body temperature (37℃) and supine position.

Video2. Velocity fields at two cross-sections in the ventricle at body temperature (37℃) and prone position.

Video3. Velocity fields at two cross-sections in the ventricle at body temperature (20℃) and supine position.

Video4. Velocity fields at two cross-sections in the ventricle at body temperature (20℃) and prone position.

Video5. Velocity fields at two cross-sections in the ventricle at body temperature (5℃) and supine position.

Video6. Velocity fields at two cross-sections in the ventricle at body temperature (5℃) and prone position.

Video7. Temperature fields at two cross-sections in the ventricle at body temperature (5℃) and supine position.
